# Saudi Hospitals Compliance With the Baby-Friendly Hospitals Initiative Evaluation Study

**DOI:** 10.7759/cureus.27914

**Published:** 2022-08-12

**Authors:** Khaled Alghamdi

**Affiliations:** 1 Pediatrics, Taibah University, Medina, SAU

**Keywords:** mothers, postnatal care, prenatal care, initiative, who guideline, unicef, baby-friendly hospitals, breastfeeding

## Abstract

Introduction

National surveys from Saudi Arabia have shown that the breastfeeding rate in Saudi Arabia is lagging behind the global recommendations. The UNICEF and WHO have launched the 10-step Baby-Friendly Hospital Initiative (BFHI) for encouraging healthcare facilities across the world to support breastfeeding in a better way. In this study, we validated the Arabic version of the self-appraisal and monitoring tool based on the BFHI as well as assessed the determinants of breastfeeding practice in Saudi Arabia.

Methods

This was an analytical cross-sectional study. We used the free validated tool-Questionnaire for Breastfeeding Mother based on the BFHI Session 4.2 Guidelines. The tool was translated and validated in Arabic. A nonprobability sample included mothers of children based on the following inclusion criteria: 1) mothers living in Saudi Arabia; 2) mothers of children aged 0-12 months. The Arabic version was modified into six parts, and the questionnaire was left open for respondents for a period of six months. Descriptive statistics were performed using the Statistical Package for the Social Sciences, version 21 (IBM Corp., Armonk, NY).

Results

The sample size was 584, and during prenatal visits, 23.6% of mothers were provided with information about skin-to-skin contact immediately after childbirth. Of these, 40% started breastfeeding immediately and 43% were encouraged to breastfeed postnatally. On discharge, 34.6% of mothers received help for feeding-related issues.

Conclusion

Our hospitals are well set to adopt the BFHI in terms of policy making and coordinated postnatal care. However, prenatal care should be more focused on promoting breastfeeding. Massive and coordinated quality improvement steps are highly indicated to completely implement the initiative.

## Introduction

National surveys from Saudi Arabia have shown that the breastfeeding rate in Saudi Arabia is lagging behind the global recommendations [1]. A study from a tertiary healthcare facility in Riyadh has revealed a breastfeeding initiation rate of 95%. However, in that study, only 1.7% of the mothers continued breastfeeding exclusively [2]. Another study involving Riyadh and Dammam populations has shown that 76% of mothers initiated breastfeeding, whereas only 37% of them practiced exclusive breastfeeding for the first six months of life of their infants [3]. In contrast, a survey involving a large sample from Al-Hassa has shown that 91.9% of newborns were breastfed. Nevertheless, only 11.4% were breastfed during the first hour [4].

In 2014, 17 cross-sectional studies were identified in a systematic review by Aljuaid et al., who aimed to assess the available data on breastfeeding in Saudi Arabia; however, they found it difficult to estimate the rate of breastfeeding because of the wide ranges in different studies; for example, the rate of exclusive breastfeeding practice ranged from 0.8% to 43.9%. Therefore, the authors concluded that no longitudinal studies have been conducted in Saudi Arabia in this regard [5].

Several scholars have studied different confounding factors that can impact breastfeeding practice [6-12]. These factors were elaborated in a systematic review by Beake et al. in 2011, who concluded that in few studies, bias was controlled while collecting data regarding breastfeeding and its confounding factors [13]. This made it difficult to monitor breastfeeding practice at the population level; few countries were successful to do so despite the huge efforts toward accurate reporting and measurement [14].

The UNICEF and WHO launched the ten-step Baby-Friendly Hospital Initiative (BFHI) to encourage health facilities worldwide to better support breastfeeding. The initiative clearly and accurately determined these 10 steps starting with hospital policy and staff competency, followed by antenatal care and care right after birth, and finally mother support with breastfeeding; supplementation; rooming-in; responsive feeding; bottles, teats, and pacifiers; and discharge [15]. The UNICEF stressed that the BFHI should continue to be implemented and that designated health facilities must be monitored and reassessed on an ongoing basis. The UNICEF developed a full set of tools for monitoring and assessing the global performance toward having the initiative implemented up to a high-quality performance to sustain its impact [16]. In this study, we validated the Arabic version of the self-appraisal and monitoring tool based on the BFHI and assessed the determinants of breastfeeding practice in Saudi Arabia.

## Materials and methods

Study design

This was an analytical cross-sectional study.

Study setting and study period

We used the free validated tool, Questionnaire for Breastfeeding Mother, based on the BFHI Session 4.2 Guidelines and Tools for Monitoring Baby-Friendly Hospitals. The questionnaire was reviewed and translated into Arabic. Subsequently, two pediatricians and one consultant of community medicine reviewed it. Subsequently, it was distributed to 50 random respondents over a one-week period. After that, it was re-evaluated by an expert statistician to assess its reliability and validity. The questionnaire was modified accordingly and distributed online to mothers in the community through different breastfeeding facilitators in different hospitals in Madinah, Jeddah, Makkah, Riyadh, and other cities.

Study population and sampling

This was a nonprobability sample involving mothers of children based on the following inclusion criteria: 1) mothers living in Saudi Arabia; 2) mothers of children aged 0-12 months. The target sample size was 800.

Data collection

The questionnaire was left open for participation over a six-month-period. The original English questionnaire consisted of the following eight parts (Table 1).

**Table 1:** Components of the English questionnaire.

Components	
1	Demographic information about the mother and her baby.
2	Pre-natal follow-up.
3	Post-natal course.
4	Skin-skin contact.
5	Feeding history.
6	Assessment of source/s of information and knowledge.
7	Breastfeeding experience.
8	Complications related to breastfeeding.

The Arabic version was modified into six parts (Table 2).

**Table 2:** Components of the Arabic questionnaire.

Components	
1	Demographic information about the mother and her baby including:
	Mother’s age, nationality, residency, education, job, father’s age. father’s education. father's job, monthly income of the family.
2	Prenatal follow-up.
	The number of antenatal visits, institute/s of antenatal care, specific questions about breastfeeding education during antennal care.
3	Postnatal course.
	Gestational age, mode of delivery, birth weight.
4	Care right after birth including: Skin-to-skin care, breastfeeding assistance and experience, supplementing, rooming-in, and responsive feeding. Bottles, teats, and pacifiers.
5	Discharge and experience after discharge.
6	Source of information.

Questions regarding knowledge were integrated within different sections to maintain the flow of thoughts and to assess the role of the 10 steps in promoting breastfeeding practice (Table 3).

**Table 3:** Knowledge assessment questions.

Assessment questions	
Section 2:	The importance of immediate skin-to-skin care. The value of rooming-in, and the risks of giving other supplements to breastfed babies during the first 6 months of life.
Section 4:	The timing and duration of the first skin-to-skin care. Breastfeeding techniques. Breast milk expression.
Section 5:	The duration of each breastfeeding session. Reinforcement of the knowledge regarding other supplements during the first 6 months of life, and reaction to mastitis, if any.
Section 6:	Direct questions about the impact of breastfeeding on reducing the risk of breast cancer.

Statistical analysis

Data were extracted from the Excel sheet and analyzed using the Statistical Package for the Social Sciences, version 21. Descriptive statistics were presented as frequencies and percentages for qualitative variables and measures of central tendency (mean).

Ethical considerations

Ethical approval was sought from the Ethics Committee of the College of Medicine, Taibah University (IRB00010413 on March 22, 2021). Informed consent was obtained from the participants as a mandatory part of the questionnaire. The privacy of data was conserved using a password-protected Excel sheet, which was saved by the principal author. All data were anonymous, and no personal data were requested or obtained.

## Results

Of the 802 respondents, 218 were excluded because they did not meet the inclusion criteria of the target population. The target age for babies was 0-1 year. The sample size was (n=584), accordingly. The demographic data are shown in Table 4.

**Table 4:** Demographic data of the Participants. SD: Standard Deviation.

Mother’s age (years)	Mean ± SD	30.3 ± 5.2
Baby’s age (months)	Mean ± SD	6.4 ± 3.7
	Frequency	Percentage
Nationality		
Saudi	522	89.4
Non-Saudi	62	10.6
City		
Makkah	35	6.0
Madinah	108	18.5
Riyadh	77	13.2
Jeddah	235	40.2
Other cities	129	22.1
Mother’s education level		
Elementary	1	0.2
Middle school	1	0.2
Secondary	73	12.5
Bachelor Degree	409	70.0
Master/Doctoral Degree	100	17.1
Mother’s work		
Employee	245	42.0
Housewife	339	58.0
Father’s education level		
Elementary	4	0.7
Middle school	7	1.2
Secondary	117	20.0
Bachelor Degree	338	57.9
Master/Doctoral Degree	118	20.2
Father’s job		
Employed	545	93.3
Unemployed	39	6.7

The number of antenatal visits was assessed. The mean number of visits was 8.3 ±4.4 SD. 91.6% of pregnancies were booked, 59.4% were under the care of obstetricians while the rest were under the supervision of other practitioners.

During prenatal visits, 23.6% of mothers were provided with information about skin-to-skin contact immediately after birth, 14.6% were provided with information on the importance of rooming-in, and 25% were educated about the risks of giving other supplements, instead of breastfeeding.

**Figure 1:**
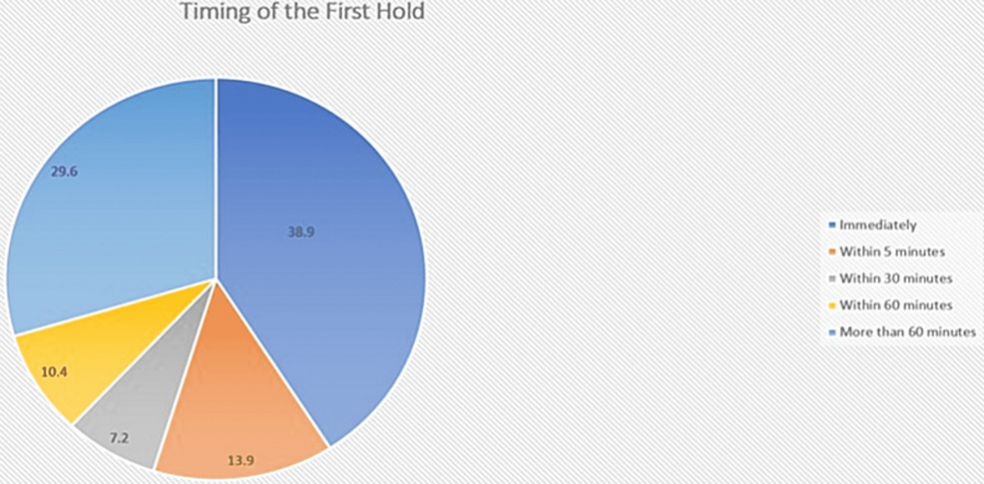
Timing of the first hold.

Regarding the postnatal course, 86% of mothers gave birth to babies at term, 61.8% delivered through vaginal delivery, 38.2% delivered by cesarean section and other modalities, and 9.1% underwent general anesthesia. The timing of the first hold is shown in Figure 1.

The reasons behind the delay in holding the baby immediately after birth were as follows; 19.1% of babies required direct medical help/observation, 9.1% of mothers were still under the effect of anesthetics, 5.6% were fully awake but with low energy, 15.5% didn't know why and 11% delay happened secondary to other reasons.

36.8% of mothers held their baby not wrapped with bare skin to allow direct ski-skin contact. 14.6% of the delay happened due to the mother’s medical condition or request. The first hold lasted less than 30 minutes in 64.2% of the study population. Upon discharge, guidance for help with feeding issues was provided to 34.6% of mothers, whereas 26.2% of mothers were involved in support groups. Staff support and detailed postnatal care are illustrated in Table 5.

**Table 5:** Detailed postnatal care.

	Frequency	Percentage
How did you hold your baby, this first time?		
Skin-to-skin	215	36.8
Wrapped without much skin contact	369	63.2
If it took more than 5 min after birth for you to hold your baby, what was the reason?		
There was no delay	213	39.8
My baby needed help/observation	102	19.1
I had been given anesthesia and wasn’t yet awake	48	9.0
I didn’t want to hold my baby or didn’t have the energy	30	5.6
I wasn’t given my baby this soon and do not know why	83	15.5
Others	59	11.0
Approximately how long did you hold your baby for this first time?		
Less than 30 min	375	64.2
30 min to <1 h	85	14.6
≥1 h	47	8.0
Can’t remember	77	13.2
During this first time when your baby was with you, did anyone from the staff encourage you to look for signs that your baby was ready to feed and offer you help with breastfeeding?		
Yes	253	43.3
No	331	56.7
Did the staff provide you with any help with breastfeeding since that first time?		
Yes	281	48.1
No	303	51.9
If yes, how long after birth was this help offered?		
Within 6 h after the baby was born	163	58.0
More than 6 h after the birth of your baby	118	42.0
Did the staff provide you with any help with positioning and attaching your baby for breastfeeding before discharge?		
Yes	348	59.6
No	221	37.8
The staff offered help, but I didn’t need it.	15	2.6
If yes, were you taught how to express (sterilize) milk?		
Yes	136	39.1
No	212	60.9
Have you tried expressing your milk yourself?		
Yes	446	76.4
No	138	23.6
If yes, was the attempt successful?		
Yes	360	80.7
No	86	19.3
Has your baby sucked on a pacifier (dummy or soother), as far as you know, while you’ve been in the maternity unit? Yes	87	14.9

In terms of knowledge, according to the answers to indirect knowledge questions, 38.8% of mothers felt that their breast milk was inadequate, and 61.3% provided feeding whenever the baby cried. When answering the direct knowledge assessment questions, 75.9% of mothers evaluated the information about the relation to breast cancer as correct, and 75.3% will continue breastfeeding even if mastitis occurs.

## Discussion

In this study, we did not evaluate Step 1 because, in Saudi Arabia, there is a Royal Decree that is already implemented by both governmental and private sectors of health care. It can be easily accessed under the name of the Law of Trading in Breastfeeding Substitutes [17].

It was clear that prenatal visits did not help much in promoting breastfeeding practice, as reflected by the percentages of providing specific information prenatally about the first hold with direct skin-to-skin contact and its value in terms of better adaptation of the newborn baby to the extra-uterine environment as well as the impact on the bonding and breastfeeding practice, the importance of rooming-in and value of such a step in making mothers more confident and excited about the breastfeeding and the other aspects of the newborn care, and the risks associated with adding supplements to breastfeeding below six months of life the period when exclusive breastfeeding unless medically contraindicated is considered the best modality of supplying the newborn with the best source of nutrition. This impression was also concluded by the authors who conducted a longitudinal study to compare the impact of the BFHI on breastfeeding in Saudi Arabia, despite the higher rates of breastfeeding in hospitals with BFHI culture; they found significant weaknesses that required strict compliance to the guidelines of the initiative [18].

In this study, the average income of the families involved was 10,000 SR (2660$) and more. Regardless of the statistical impact of such information as it might reflect a biased sampling, it has been concluded by authors who evaluated the BFHI in 35 countries of different income situations; they concluded that there is a huge response indicating the readiness to implement the initiative in neonatal wards globally [19].

Adopting and supporting the BFHI is a major step toward better standards of care for newborns. Studies that demonstrated that the BFHI is ineffective in promoting breastfeeding practice were hampered by weak designs and study limitations, as concluded in a systematic review conducted to evaluate the impact of the initiative [20].

When it comes to specific evaluation of different steps of the evaluation, we found that the postnatal performance is better than that in the prenatal period, as demonstrated in the steps of immediate postnatal care and upon discharge. However, we could not find a more effective step than the others. This exactly matches the outcome of a systematic review that concluded that the lack of standardization and variable study designs and multiple confounders limited the ability to recommend any single intervention as the most effective. This conclusion highlights the importance of validating the Arabic tool and making it a standard of evaluation in future assessments [21]. Finally, although most couples who participated in this study were highly educated when it came to the real practice and knowledge assessment, the mothers did not indicate a high level of knowledge on breastfeeding.

Limitations and future directions

This was a cross-sectional study that has its own design limitation; there is a chance of a recall bias, and it might not be the best option to assess the behavior of mothers toward breastfeeding. However, this study was designed to evaluate the process itself in its different steps.

## Conclusions

Hospitals in Saudi Arabia are well set to adopt the BFHI in terms of policymaking and coordinated postnatal care. However, prenatal care is not as effective as these particular steps.

The number of antenatal visits in this study is considered higher than the standards of basic regular antenatal care. However, it is recommended that antenatal visits must put more effort into focusing on breastfeeding practice promotion. That includes formal breastfeeding sessions directed to both parents, elaborating the importance of skin-skin contact as early as possible, encouraging mothers to get immediately engaged in the care of newborns, and enhancing staff involved in delivery wards to acknowledge, address and maintain such steps in order to improve the outcome of breastfeeding promotion and support.

The validated Arabic version of the evaluation tool developed and utilized in this study is a subject of further implementation and modification if indicated. Massive, coordinated quality improvement steps to completely implement the initiative are highly indicated. Further high-quality randomized controlled trials are needed to evaluate such an initiative.

## Appendices

**Table 6:** The Ten-Steps Baby-Friendly Hospitals Initiative

Ten steps	
1	1a. Comply fully with the International Code of Marketing of Breast-milk Substitutes and relevant World Health Assembly resolutions. 1b. Have a written infant feeding policy that is routinely communicated to staff and parents. 1c. Establish ongoing monitoring and data-management systems.
2	Ensure that staff have sufficient knowledge, competence and skills to support breastfeeding.
3	Discuss the importance and management of breastfeeding with pregnant women and their families.
4	Facilitate immediate and uninterrupted skin-to-skin contact and support mothers to initiate breastfeeding as soon as possible after birth.
5	Support mothers to initiate and maintain breastfeeding and manage common difficulties.
6	Do not provide breastfed newborns any food or fluids other than breast milk, unless medically indicated.
7	Enable mothers and their infants to remain together and to practice rooming-in 24 hours a day.
8	Support mothers to recognize and respond to their infants’ cues for feeding.
9	Counsel mothers on the use and risks of feeding bottles, teats and pacifiers.
10	Coordinate discharge so that parents and their infants have timely access to ongoing support and care.
